# Incidence and Outcomes of in-Hospital Cardiac Arrest and Cardiopulmonary Resuscitation in the Kidney Replacement Therapy Population: Protocol for a Linked National Disease Registry Study

**DOI:** 10.2196/83272

**Published:** 2026-05-26

**Authors:** Robert McLaren, Barnaby Hole, Zofia Tuharska, Maria Pippias

**Affiliations:** 1 Population Health Sciences, Bristol Medical School, University of Bristol, Canynge Hall 39 Whatley Road, Bristol, BS8 2PS, United Kingdom, 64 02904509312; 2 Renal Unit, North Bristol NHS Trust, Bristol, United Kingdom; 3 Homerton University Hospital, London, United Kingdom

**Keywords:** cardiopulmonary resuscitation, CPR, dialysis, transplant, kidney replacement therapy, end stage renal disease, chronic kidney disease, CKD, survival

## Abstract

**Background:**

Every patient receiving kidney replacement therapy that is, hemodialysis, peritoneal dialysis or a kidney transplant, should have an advanced treatment escalation plan. This should include a decision on cardiopulmonary resuscitation (CPR) status. The existing literature suggests kidney replacement therapy recipients who undergo in-hospital CPR have outcomes similar to the general population. However, these data originate exclusively from North America and Taiwan, do not include transplant recipients, and neurological outcomes are poorly reported. This paucity of evidence influences how kidney replacement therapy recipients, their families and care providers make informed discussions on CPR status and may leave kidney replacement therapy recipients vulnerable to false assumptions of poor suitability.

**Objective:**

Our primary objective is to calculate the incidence and survival rates of in-hospital CPR in the kidney replacement therapy population in England over a 10-year period (2012‐2022). Further, we aim to compare the characteristics of survivors versus nonsurvivors, and describe neurological outcomes.

**Methods:**

We will conduct a retrospective cohort study of patients receiving kidney replacement therapy aged≥28 days in England who underwent in-hospital CPR between 2012 and 2022. Individuals receiving kidney replacement therapy will be identified as reported to the United Kingdom Renal Registry (UKRR). Case matching will occur between The UKRR and The Intensive Care National Audit & Research Center (ICNARC) which holds the UK National Cardiac Arrest Audit (NCAA) database. The annual cumulative incidence of in-hospital CPR events will be ascertained by kidney replacement therapy modality. Survival and neurological outcomes will be reported aligned with international data reporting standards. The characteristics of survivors versus nonsurvivors and individuals with favorable versus individuals with nonfavorable neurological outcomes will be compared to identify individual or situational factors that associate with outcomes.

**Results:**

As of February 2026, ethical approval has been granted by The South West - Cornwall & Plymouth Research Ethics Committee. Section 251 support has been granted to allow the processing of confidential information (limited to date of birth, NHS number and date of kidney replacement therapy start) without consent between the UKRR and ICNARC, and date of death only between ICNARC and the University of Bristol Researchers. This excludes individuals who have opted out of their data being used for research purposes by the UKRR.

**Conclusions:**

By linking registry data this study will provide a whole-of-population approach to describing the incidence and outcomes of in-hospital CPR in the English kidney replacement therapy population. This will provide patients, their loved ones, and care providers data on the likelihood of in-hospital CPR success which will aid the advanced care planning in this setting.

## Introduction

Cardiopulmonary resuscitation (CPR) is an emergency intervention to restore cardiac function following a cardiac arrest. It involves manual chest compressions, intravenous medications and, where indicated, defibrillation [1]. In the absence of a ‘do not attempt resuscitation order (‘DNACPR’), CPR is usually initiated in the event of witnessed cardiac arrest [2]. Some patients may decide upon a DNACPR due to the burden of multiple medical conditions and to avoid invasive medical interventions which may provide little benefit. For patients and their care providers to make informed decisions regarding DNACPR status, data relating to the likely outcomes following resuscitation are needed.

In the United Kingdom, the survival to discharge rate following in-hospital cardiac arrest is 24% [3], meaning 76% of those who receive CPR either do not experience a return of spontaneous circulation, or do, but die before leaving hospital. Approximately 98% of survivors have a ‘favorable’ neurological function, defined by the cerebral performance category (CPC) grade one or two. This encompasses normal to moderate cerebral dysfunction, compatible with at least part-time work in a sheltered environment, and independent activities of daily life (Table 1) [3]. The 2% with ‘unfavorable’ outcomes are dependent upon others for daily support. Survivors of CPR may sustain significant injury during resuscitation such as rib and sternal fractures, pneumothorax and liver injury [4]. They also experience psychological distress, and a significant number develop post-traumatic stress disorder [5]. Out-of-hospital cardiac arrest has markedly poorer outcomes in comparison, with a 30-day survival of 9.5% in the United Kingdom, and international reporting standards consider in—hospital and out-of-hospital CPR separately [6-8].

**Table 1.** Cerebral performance category: table presenting the five categories of cerebral performance with attached description. Adapted from [3].

Score	Category	Description	Favorable/unfavorable
1	Normal	Conscious, alert, able to work and lead a normal life May have minor psychologic or neurologic deficits (eg, mild dysphasia, nonincapacitating hemiparesis, minor cranial nerve abnormalities)	Favorable
2	Moderate disability	Conscious, with sufficient cerebral function for part-time work in sheltered environment and independent activities of daily life (eg, dress, travel by public transportation, prepare food) May have hemiplegia, seizures, ataxia, dysarthria, dysphasia, or permanent memory or mental changes	Favorable
3	Severe disability	Conscious, but dependent on others for daily support; has at least limited cognition This category includes a wide range of neurologic dysfunction, from patients who are ambulatory but have severe memory disturbances or dementia that precludes independent existence to those who are paralyzed and can communicate only with their eyes (as in the locked-in syndrome).	Unfavorable
4	Unconscious	Unconscious, unaware of surroundings, no cognition; no verbal or psychologic interaction with environment	Unfavorable
5	Brain death	Meeting criteria for brain death or dead by traditional criteria	Unfavorable

Historically, individuals receiving kidney replacement therapy ie, hemodialysis, peritoneal dialysis or a kidney transplant, have been depicted as poor candidates for CPR [9]. This group are known to be at high risk of cardiac arrest [10] and may experience unique risk factors. Examples include end-organ damage secondary to diabetes mellitus, hyperkalemia and hypervolaemia due to end stage kidney disease, and intradialytic fluid shifts and changes in serum electrolyte levels that occur during dialysis [11,12]. However, some of these aetiologies are reversible causes of cardiac arrest, which may mean that those with kidney failure are simultaneously at high risk for cardiac arrest, and—in some circumstances—more likely to experience favorable CPR outcomes [11,12].

We conducted a systematic literature review (manuscript currently under review) which found that survival to discharge from hospital after in-hospital or in-dialysis unit CPR amongst patients receiving kidney replacement therapy was 18%‐30%. These outcomes are broadly comparable with those of the general inpatient population. A recent study of CPR amongst hemodialysis recipients confirmed the association between dialysis sessions and cardiac arrest, and found favorable outcomes, with 30% surviving to hospital discharge—likely due to early CPR and the presence of shockable dysrhythmias [10]. In our systematic review, standardized neurological outcomes were only reported in two studies and kidney transplant recipients were not represented. None of the included studies differentiated between individuals receiving acute and chronic kidney replacement therapy, nor time since kidney replacement therapy initiation, despite the higher rates of death early after kidney replacement therapy initiation [13]. The eleven identified studies were conducted in the United States of America, Canada and Taiwan. Regarding CPR outcomes, these data must be understood in the light of the population at risk and the practices surrounding CPR decision-making, both of which likely to vary between countries.

The limited geographical range of data, paucity of neurological outcome reporting, and heterogeneity of survival outcome measures leaves a substantial knowledge gap. More comprehensive and international data describing the survival and functional outcomes for people with kidney failure who receive in-hospital CPR will inform patients and their care providers making treatment escalation plans. We present a protocol for a retrospective observational cohort study which aims to describe the incidence and outcomes following in-hospital CPR in the English kidney replacement therapy population. Our objectives are to:

1. Determine the annual incidence of in hospital cardiac arrest treated with CPR in the prevalent English kidney replacement therapy population
2. Determine the cumulative incidence of in hospital cardiac arrest treated with CPR in the incident English kidney replacement therapy population
3. Calculate the probability of survival following in hospital cardiac arrest treated with CPR at pre-determined time points: return of spontaneous circulation (ROSC), survival to 30 days, discharge from hospital, or survival to 1 year, adjusted for confounders using multivariable logistic regression, at each of these time points. The characteristics of survivors and nonsurvivors of in-hospital CPR at the aforementioned time points will also be described
4. Determine the cerebral performance category (CPC) at discharge of kidney replacement therapy recipients who survived in hospital CPR, comparing the characteristics of those who had ‘favorable’ neurological outcome (CPC 1 or 2) versus those who did not (CPC 3‐5)

## Methods

### Overview

This protocol outlines a retrospective observational cohort study of in-hospital cardiac arrest in the English kidney replacement therapy population from 2012‐2022. This will be facilitated by a one-off data linkage between two UK-based registries, the UK Renal Registry (UKRR) and the Intensive Care National Audit & Research Center (ICNARC), which holds the National Cardiac Arrest Audit (NCAA) database. The Standardized Protocol Items Recommendations for Observational Studies was used to aid protocol development and is included in Checklist 1 [14].

### Datasets for Linkage

The United Kingdom Renal Registry is the UK Kidney Association’s registry of people receiving nephrology specialist care [15]. It receives data from National Health Service (NHS) kidney services in England, Wales and Northern Ireland, which are mandated to provide information regarding all individuals receiving acute or chronic kidney replacement therapy, under their care. Since nearly all kidney replacement therapy is provided by NHS centers in England, the UKRR is believed to have almost complete coverage of individuals receiving long-term kidney replacement therapy for kidney failure.

The NCAA is the national clinical audit of in-hospital cardiac arrests in the UK. It holds data on cardiac arrests attended to by in-hospital resuscitation teams for all individuals aged ≥28 days [16]. Criteria for inclusion is “any resuscitation event, commencing in-hospital, where an individual (excluding neonates) receives chest compression(s) and/or defibrillation and is attended by the hospital-based resuscitation team (or equivalent) in response to a 2222 call” [16]. Current coverage includes approximately 93% of acute hospitals in England [17]. The NCAA does not include cardiac arrests that occur within intensive care units as the hospital resuscitation team does not attend such events. Further, cardiac arrests occurring in satellite dialysis units are only included where the unit is covered by an inpatient cardiac arrest team.

### Data Linkage Process

Case-matching between the two registries will be conducted through the transfer of encrypted NHS numbers, dates of birth and date of commencing kidney replacement therapy from the UKRR to ICNARC.

- Step 1: The UKRR will provide ICNARC with a list of encrypted NHS numbers, dates of birth, date of commencing kidney replacement therapy, plus a randomly generated study-ID for the population receiving kidney replacement therapy between 2012‐2022.
- Step 2: Using a corresponding encryption/decryption key, ICNARC will identify individuals also present in their NCAA database.
- Step 3: For individuals who are matched, a secure platform will be used to send the requested data fields (see Multimedia Appendix 1), uniquely identified by the encrypted NHS numbers, with the study-ID number to the University of Bristol researchers.
- Step 4: the University of Bristol researchers will inform the UKRR which patients have been identified as having a CPR event from their dataset by sending the study-ID and date of CPR back to the UKRR. This will allow the UKRR to identify the corresponding individuals within their dataset.
- Step 5: The UKRR will also send the corresponding requested data fields of the prevalent kidney replacement therapy population to the University of Bristol researchers with the study-ID number but not the encrypted NHS number or full date of birth to allow linkage to the NCAA dataset. This method will ensure that at no point does the encrypted NHS number or full date of birth flow with clinical data and that the University of Bristol Researchers do not have access to the encrypted NHS number or full date of birth (month/year only).

**Figure 1.**
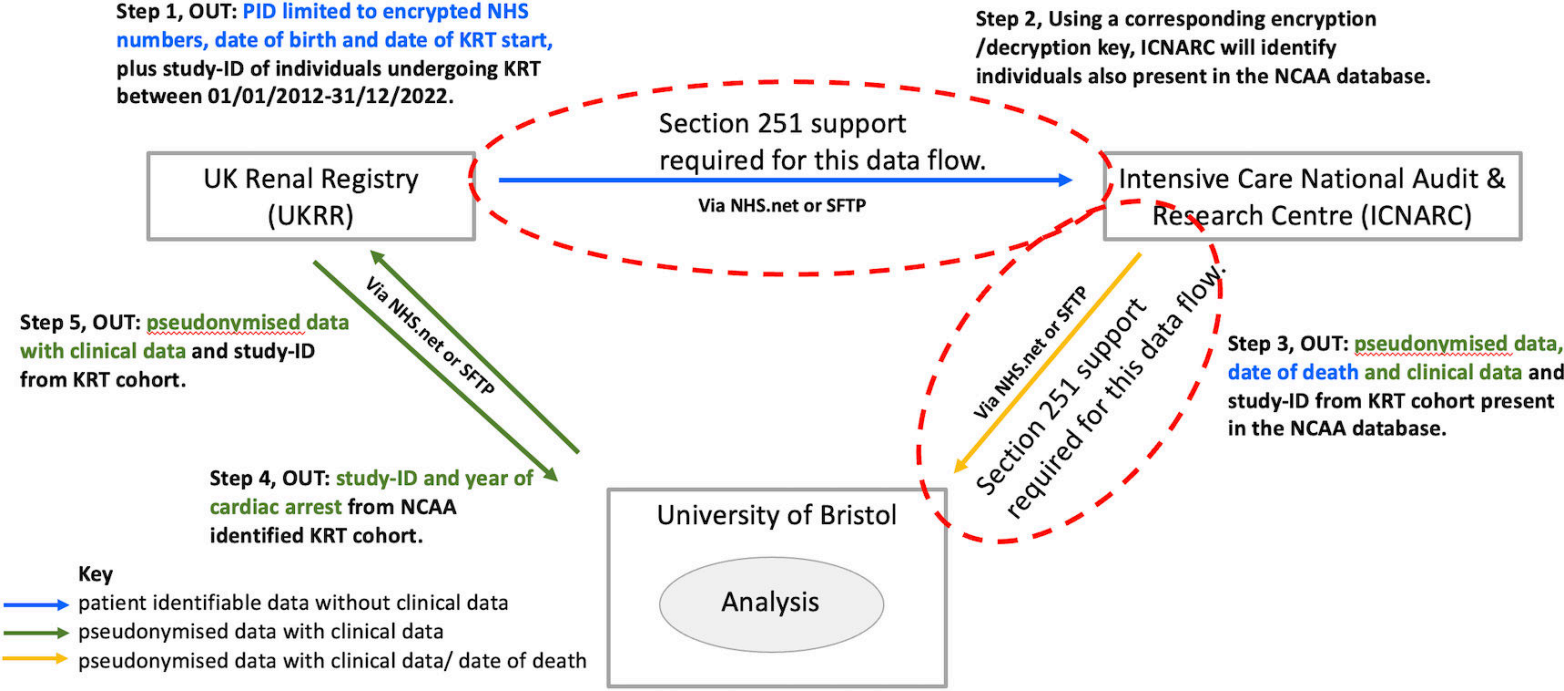
Proposed data flow for the study ‘Incidence and outcomes of in-hospital cardiac arrest in the United Kingdom kidney replacement therapy population. KRT kidney replacement therapy; PID: Patient Identifiable Data; NCAA: national cardiac arrest audit; SFTP: secure file transfer protocol.

The data will be securely stored within the University of Bristol, with access to the physical server will be restricted to IT services and Security staff employed by the University. University of Bristol researchers will not have access to identifiable information of included or excluded individuals, except for the date of death. The data flow is summarized in Figure 1. The total number of patients in the English kidney replacement therapy cohort who receive in-hospital CPR is unknown and will be an output of this work.

### Study Population

Eligible cases will be individuals≥28 days old (i) reported to the UKRR by an English Kidney center, (ii) as having received dialysis (hemodialysis or peritoneal) or a kidney transplant, (iii) who underwent in-hospital CPR for cardiac arrest between Jan 1st, 2012, and Dec 31st, 2022. Acute and chronic dialysis will be defined as per the UKRR [18]. Dialysis initiation is classified as ‘chronic’ from the date of start in the UKRR data if an individual is receiving dialysis 90 days after starting, or if the clinician caring for the patient judges the kidney failure to be irreversible and codes it as ‘chronic’ before day 90. Transplant will be classified as individuals living with a functional kidney transplant. Patients who experience cardiac arrest within an intensive care unit will not be included, as they are not recorded in the NCAA. Further, patients receiving hemofiltration will be excluded as this occurs in the intensive care unit setting. Cardiac arrests occurring in satellite dialysis units will only be included where the unit is covered by an inpatient cardiac arrest team. As both adult and pediatric patients will be included, separate reporting of all baseline and outcome data will be presented for the 28 days-17 years and ≥18 years groups. Further age-based subgroup analyses within the 28 days–17 years cohort will be performed if sample size permits.

### Baseline Variables

These will include but may not be limited to; age, sex, ethnicity, socioeconomic deprivation quintile, primary renal disease, diabetes status, malignancy status, kidney replacement therapy modality and kidney replacement therapy duration (See Multimedia Appendix 1 which lists all variables requested from the two Registries).

### Objective 1 – Annual Incidence of in-Hospital CPR in the Prevalent Kidney Replacement Therapy Population

The annual incidence of in-hospital CPR will be calculated as the number of recorded in-hospital CPR events within the annual cohort of prevalent dialysis and kidney transplant recipients, expressed as CPR events per 1000 kidney replacement therapy recipients per annum between 2012 to 2022. The size of the prevalent population will be calculated on 31st December of every year. Incidence will be expressed within clinicodemographic strata, including age, sex, kidney replacement therapy modality, location of cardiac arrest (ward vs dialysis unit) and dialysis status (acute vs chronic).

### Objective 2 – Cumulative Incidence of in-Hospital CPR in Incident Kidney Replacement Therapy Population

Cumulative incidence of in-hospital CPR events per 1000 incident kidney replacement therapy recipients will be calculated as the number of recorded in hospital CPR events for either incident dialysis or incident kidney transplant recipients. The day of commencing kidney replacement therapy (either dialysis or transplantation) will be the starting point, and individuals will be followed up to the point of either the outcome of interest (first CPR) or censored for a change of modality ie, to kidney transplantation or dialysis respectively, death or end of the follow-up period set at 31-12-2023 or loss to follow-up. The cumulative incidence will be adjusted for confounders which have been determined a priori ie, age, sex, primary kidney disease, diabetes status.

### Objective 3 – Survival Following in-Hospital CPR in the Prevalent Kidney Replacement Therapy Population

Crude survival outcomes for those who survived CPR will be determined by drawing Kaplan-Meier curves and calculating the median survival time post CPR. Log-rank test for survival by treatment modality, sex, and age group will be performed.

Multivariable logistic regression will be used to determine the odds of survival to ROSC, 30 days, discharge and 1 year. The output of this model will also be presented by kidney replacement therapy modality, dialysis status (acute vs chronic), location of cardiac arrest (ward vs dialysis unit), age group, primary renal disease and diabetes. The model will be adjusted by kidney replacement therapy duration, age (when not stratified by age), sex (when not stratified by sex), primary renal disease (when not stratified by primary renal disease) and diabetes status (when not stratified by diabetes status) as these are established predictors of CPR survival [19].

For all survival analysis, the date of first CPR event will be the start date. Individuals will be followed until the event of interest ie, ROSC, survival to 30 days, survival to discharge, survival to 1 year or death or loss to follow up. If more than one CPR event occurs within a single hospital admission only the first CPR event will be considered. Further CPR events occurring in subsequent admissions for the same individuals will be treated as separate events. We will include a random intercept in the generalized linear mixed model, using a binomial distribution, to account for the dependency of these subsequent admissions and CPR events.

The characteristics of individuals who survived to discharge from hospital will be compared with those of individuals who did not. Continuous variables and categorical variables of survivors and nonsurvivors will be compared using the Mann-Whitney *U* test and Chi-squared test, respectively.

### Objective 4 – Neurological Outcome Following in-Hospital CPR

Characteristics of those who survived with favorable neurological outcome and those with unfavorable neurological outcomes (defined by a cerebral performance category of 3 or greater ie, having at least severe disability dependent in activities of daily living) will be presented. Continuous variables and categorical variables will be compared using the Mann-Whitney *U* test and Chi-squared test respectively.

Multivariable logistic regression will be used to determine the odds of favorable versus nonfavorable CPC. Favorable CPC will be defined as a CPC of 1 or 2. CPC 1 is described as good cerebral performance with none/mild neurological or psychiatric deficit, while CPC 2 represents moderate cerebral disability with sufficient cerebral function to allow for independent activities of daily living. The output of this model will also be presented by treatment modality, dialysis status (acute vs chronic), location of cardiac arrest (ward vs dialysis unit), age group, primary renal disease and diabetes. The model will be adjusted by kidney replacement therapy duration, age (when not stratified by age), sex (when not stratified by sex), primary renal disease (when not stratified by primary renal disease) and diabetes status (when not stratified by diabetes status) as these are established predictors of CPR survival [19].

Outcomes will be presented by unit if sample size allows. Units will only be presented anonymously in any subsequent research output (most likely as funnel plots) which will help to identify centers that outlie expected variation.

### Minimum Sample Size and Predicted Event Rate

It is not possible to predict an event rate as CPR incidence in this population has never been studied before in Europe. Studies conducted in other health systems who did not include transplant recipients report rates of 1.4‐5.08 events per 1000 admission days [20,21]. One of the objectives of this study is to report on the event rate in the study population.

### Risk of Bias

The cerebral performance category is internationally recognized for describing neurological outcome following cardiac arrest [8]. However, no formal standardization training is offered, and this may allow inter-rater variance and hence misclassification bias. Further, socioeconomic deprivation will be ascertained using 2015 national census data [22], it is possible Individuals’ socioeconomic status has changed since data was last collected, this may also lead to misclassification bias.

### Confidentiality and Re-Identification Risk

Where any strata or cell in the report of these analyses includes fewer than five individuals, the cell will be expressed as 0‐5 to reduce the risk of re-identification. Any breeches in confidentiality will be immediately reported to the University of Bristol governance team and dealt with in line with institutional policy.

### Missing Data and Quality Assurance

A basic descriptive analysis will initially be conducted to look at the quality of data including proportion of missing data. Missingness indicators will be examined in relation to observed covariates to assess the plausibility of a missing at random mechanism. If data are found to be missing at random and <30% missing, multiple imputation will be used to impute missing data. Missing data will be addressed using multiple imputation by chained equations, including all variables in the primary analyses and predictors of missingness and outcome, with estimates combined using Rubin’s rules [23]. Complete-case analyses will be conducted as a sensitivity analysis. If the data are missing not at random, we will only use observed data, and report on the extent of the missing data and the associated limitations.

### Training of Investigators

All investigators involved directly in data analysis will complete mandatory data safety training as per the University of Bristol guidelines. Lead investigator MP will oversee all analysis and ensure accuracy.

### Availability of Data and Full Protocol

Due to the sensitive nature of the data involved, it will not be possible to make the data publicly available following publication. A full protocol will be available once study data is acquired.

### Ethical Considerations

Approval was granted on June 26th, 2024 by the Health Research Authority South West - Cornwall & Plymouth Research Ethics Committee [24]. A research application under Regulation 5 of the Health Service (Control of Patient Information) Regulations 2002 (’section 251 support’) to process confidential patient information without consent was made to the Health Research Authority and with support granted on September 19th, 2024 (CAG reference: 24/CAG/0087). This support covers the flow of patient identifiable information (ie, NHS number, date of kidney replacement therapy start and date of birth) from the UKRR to ICNARC and the flow of date of death from ICNARC to the University of Bristol. The UKRR has screened research cohorts against NHS England National Data Opt-Out portal prior to use or transfer of data intended for research purposes and removes any opted-out patients from the cohort. Information regarding this study is available on the UKRR website [25].

### Patient and Public Involvement

Our study proposal was reviewed and approved by three patient groups; the UKRR’s patient group reviewed the study proposal, a public panel reviewed our application as part of the local funding process and agreed on the positive impact the project will have on the NHS, and the patient group in the Bristol Kidney Health Integration Team also agreed on the positive impact this project would have on patient care.

## Results

As of February 2026, ethical approval and Section 251 support has been granted. The UKRR will share with ICNARC the variables required to link to the NCAA database. We now await the data sharing agreement to be approved by our institution to allow data release. A timeline of study progress is presented in Table 2.

**Table 2.** Study timeline: Table displaying key dates of project progress and expected dates of future progression.

Progress	Achieved or expected completion
Initial application for registry data	02/2022
Funding acquired	03/2024
Ethical approval granted	06/2024
Section 251 support granted	09/2024
Data transfer to university server	Expected 04/2026
Completion of analysis	Expected 01/2027
Submission of manuscript for peer-reviewed publication	Expected 04/2027

## Discussion

### Anticipated Results

We expect principal findings of incidence, survival and neurological outcomes may be similar to those reported by our systematic review on this topic (awaiting publication). It identified six studies reporting outcomes of in-hospital CPR in this cohort. Incidence rate was reported by two studies of 1.4‐5.08 events per 1000 admission days [20,21] . Survival to discharge following in hospital CPR in this population to be 18%‐30% [20,21,26-29]. Neurological outcomes were poorly reported, only one study reported cerebral performance category, finding 69% of patients had a performance of 1‐2, classified as ‘good’ [27]. However, our study may differ to those published in that it will be the first to investigate this issue outside of North America or Taiwan. The use of a novel setting is key as culture influences DNACPR usage [30], and the case mix of individuals receiving kidney replacement therapy differs across the world [31]. Further it will also be the first to incorporate kidney transplant recipients and pediatric patients.

### Strengths and Limitations

The strengths of this study include its whole-of-population approach with the use of dedicated population level data registries. This will allow for accurate identification of eligible patients, as well as thoroughly reported outcome data presented as per internationally accepted Utstein template [8]. Further, the study explores an area of healthcare that we have demonstrated is important to patients and could directly impact how they are treated.

The limitations of this study are that sample size and extent of missing data cannot be predicted due to a lack of consistent information on the event numbers for in-hospital CPR, this is one of our objectives. The study will be unable to capture patients who have opted-out of registry data collection and this may result in nonrandom missing data. A key limitation is that ICNARC only collects data on CPR events occurring within the inpatient ward and some satellite dialysis unit settings and so our findings will not be generalized to all CPR events. One of our future recommendations will likely be the development of a study describing out-of-hospital CPR events, with the use of an appropriate registry such as The Out-of-Hospital Cardiac Arrest Outcomes Registry [32]. Only outcomes for those individuals who are deemed suitable to receive and choose to remain “for” CPR will be included. It is vital that these selection effects do not mislead readers, clinicians or patients by exaggerating the positive outcomes following cardiac arrest for people with kidney failure. Providing survival information on in-hospital CPR events will nevertheless provide valuable information for individuals and their families. This may be especially relevant for individuals felt unlikely to survive in-hospital CPR, for whom prognosis following out of hospital CPR is likely to be especially poor

### Conclusions

This study will describe in-hospital CPR incidence, survival and neurological outcomes for the English kidney replacement therapy population for the first time. This information will benefit patients by providing them, their loved ones and care providers information to make evidence-based decisions on CPR status whilst a hospital inpatient. The findings will provide objective evidence of the suitability of kidney replacement therapy recipients for CPR in this setting.

### Dissemination Plan

The dissemination plan for this study, includes preparing a manuscript for submission to a peer reviewed journal. An abstract will also be submitted for presentation at a major international conference. Authorship will be determined by International Committee of Medical Journal Editors guidelines [33]. This work is highly relevant to patient decision making and advance care planning, hence a program of patient and public involvement work is being developed to build upon what has already been completed. This will include a lay article being prepared for submission to the patient magazine ‘Kidney Matters.’

### Future Directions

Our study will provide patients receiving kidney replacement therapy with objective evidence of their likely outcomes following CPR. The logical next step in this process is to explore patient and care giver experience of the advanced care planning process. This will inform the medical community how kidney care services can best support patients to use our results, as well as their lived experiences and preferences, to make holistic and appropriate advanced care planning directives. Further, it would be valuable to conduct a study exploring out of hospital CPR events, as we have described above.

## Supplementary material

10.2196/83272 Multimedia Appendix 1 Complete list of variables requested from respective registries.

10.2196/83272 Checklist 1 Standardized Protocol Items Recommendations for Observational Studies (SPIROS) checklist.

## Abbreviations

CPC: cerebral performance category
CPR: cardiopulmonary resuscitation
DNACPR: do not attempt resuscitation order
ICNARC: The Intensive Care National Audit & Research Centre
NCAA: National Cardiac Arrest Audit
NHS: National Health Service
ROSC: return of spontaneous circulation
UKRR: United Kingdom Renal Registry
