# Supplementary material for: Incidence and Outcomes of in-Hospital Cardiac Arrest and Cardiopulmonary Resuscitation in the Kidney Replacement Therapy Population: Protocol for a Linked National Disease Registry Study
Source: JMIR Res Protoc. 2026 May 26;15:e83272. doi: 10.2196/83272 (PMC13211596; doi:10.2196/83272)
Supplement: Multimedia Appendix 1 [file resprot-v15-e83272-s001.pdf]

Multimedia Appendix 1: Complete list of variables requested from respective registries.

Legend: table presenting all variables requested from the National Cardiac Arrest Audit and United Kingdom Renal Registry.

National Cardiac Arrest Audit	United Kingdom Renal Registry
Date of birth (month/year) /Estimated age	Year of birth
Sex	Sex
Ethnicity	Hospital centre code
Date of admission to/attendance at/visit to your hospital	Index of multiple deprivation from Postcode
Reason for admission to/attendance at/visit to your hospital	Ethnicity
Date/Time of 2222 call	Date start treatment
Status at team arrival	Treatment modality code
Location of arrest	Date of kidney transplant
Presenting/first documented rhythm	Date end treatment
Date/Time resuscitation started	Date of kidney transplant failure
Date/Time resuscitation stopped	Date of death
Reason resuscitation stopped at end of team visit	Cause of death
Do not attempt cardiopulmonary resuscitation status	Primary renal disease code
Transient post-arrest location	Vascular access
Post-arrest location	Diabetes
Status at discharge from your hospital	Date diabetes diagnosed
Sedated at discharge from your hospital	Malignancy Yes / No
Modified Rankin Scale (adult) or Paediatric Cerebral Performance Category (paediatric) at discharge from your hospital	Malignancy - date first diagnosed
Method used to assess neurological status at discharge from your hospital	Malignancy site, first primary site only
Date of discharge from your hospital	Donor type of renal transplant
Date/Time of death	Transplant recipients only: lab calculated estimated glomerular filtration rate
Total number of admissions to your hospital	Transplant recipients only: date of lab calculated estimated glomerular filtration rate
Total number of 2222 calls solely for cardiac arrest	
