# Supplementary material for: Incidence and Outcomes of in-Hospital Cardiac Arrest and Cardiopulmonary Resuscitation in the Kidney Replacement Therapy Population: Protocol for a Linked National Disease Registry Study
Source: JMIR Res Protoc. 2026 May 26;15:e83272. doi: 10.2196/83272 (PMC13211596; doi:10.2196/83272)
Supplement: Checklist 1 [file resprot-v15-e83272-s002.pdf]

☑ SPIROS

Standardized Protocol Items Recommendations for Observational Studies

Table 1: SPIROS 2023 Checklist: Recommended Items to address in the observational study Protocol and related documents.

Section / Item	Item Number		Description
**Part A: General information**			
Title	1	☑	Descriptive title Identifying study design in the title
Protocol version	2	☑	Version or amendment number with date and summary of the changes
Protocol summary	3	☑	An informative and balanced summary of the study protocol
Sponsor and funder details	4	☑	Name of Sponsor and funder and types of financial, material, and other support
Conflict of interest statements	5	☑	Statement about any financial and other competing interests for principal or co-investigators for the overall study.
Investigators name	6a	☑	Names of the principal and co-investigators
Affiliation of investigators	6b	☑	Affiliated institutions of the investigators
Principal researcher/s contact detail	6c	☑	Name, e-mail address, affiliation of principal researcher
**Part B: Introduction**			
Background of the study	7a	☑	Description of research question and scientific background of the study
Review of prior research	7b	☑	Summary of relevant existing research (published or unpublished)
Rationale of study	7c	☑	Justification for conducting the study
Aim	8a	☑	Broader aims and overall objective
Objective/s of the study	8b	☑	Primary and secondary objective/s including any prespecified hypothesis (if applicable).
	8c	☑	Specify whether the intention is to (a) estimate causal effects, (b) predict outcomes, or (c) simple description.
**Part C: Methods**			
Study design	9a	☑	Description of study design (case control, cross-sectional or cohort) and type of study (retrospective cohort study, Prospective cohort study etc)
Study setting	9b	☑	Description of the study setting (e.g., community-based, hospital based) and detail of precise locations of the study sites.
Study schedule	10a	☑	Description of the expected schedule of the study including relevant dates, expected periods of recruitment/survey, exposure, follow-up, and data collection.
	10b	☑	Figure (Study schematic/flow-chart) or table describing expected time frame for each step including trainings, data collection, follow-up, analysis and reporting etc.

Section / Item	Item Number		Description
Sample size	11	☑	Estimation of minimum sample size required for the study with justifications including clinical and statistical assumptions supporting any sample size calculations.
Sampling procedure	12	☐ NA	Detailed description of the sampling frame and sampling strategy (simple random, stratified random, cluster, systematic etc.)
**Participant selection**			
Participant selection for cohort study	13a	☑	Description of inclusion and exclusion criteria, and the source and methods of participant selection (exposed and unexposed). For matched cohort studies, give matching criteria and number of exposed and unexposed.
Participant selection for case-control study	13b	☐ NA	Description of inclusion and exclusion criteria, and the source and methods of case ascertainment and control selection. Give the rationale for the choice of cases and controls. Give diagnostic criteria for identifying cases (if applicable). For matched case-control studies, give matching criteria and the number of controls per case.
Participant selection for cross-sectional study	13c	☐ NA	Description of the inclusion and exclusion criteria, and the source and methods of participant selection.
Variables	14a	☑	Detailed description of all important baseline and outcome variables to be analysed, exposures, predictors, potential confounders, and effect modifiers. Give diagnostic criteria, if applicable.
Data sources/measurement	14b	☑	For each variable of interest, give sources of data and details of assessment /measurement methods. Describe comparability of assessment methods if there is more than one group.
Data collection and management	15a	☑	Plans for assessment and collection of outcomes, baseline, follow up and other study related data.
	15b	☐ NA	Description of data collection methods e.g., online survey, Household survey, paper based or electronic data capture etc.
	15c	☐ NA	Any related processes to promote data quality during data collection (e.g., duplicate measurements, training of assessors, validation method)
	15d	☐ NA	Description of study instruments (e.g., questionnaires, data collection forms) along with their reliability and validity, if known. Reference to where data collection forms can be found, if not in the protocol.
	15e	☑	Plans for data entry, coding, security, and storage, including any related processes to promote data quality (e.g., electronic data capture, double data entry; range checks for data values, random cross-checking of electronic data with the source documents).

Section / Item	Item Number		Description
	15f	☑	Reference to where details of data management procedures can be found, if not in the protocol.
Blinding procedure (if blinded study)	16	☐ NA	Description of blinding procedure (if applicable) reporting Who will be blinded (e.g., investigator blinded for disease status when measuring exposure in case-control study) and methods to ensure blinding and unmasking of blinding if required.
Potential bias	17	☑	Description of any potential biases and plan to minimize those potential sources of biases.
Statistical analysis plan	18	☑	Detailed description of methods for analysing and presenting primary/secondary outcomes and any additional analysis (e.g. analyses of subgroups and interactions, and sensitivity analyses). Give reference to the where other details of the statistical analysis plan can be found, if not in the protocol.
Handling of missing data	19	☑	Detailed description of methods to handle missing data (e.g. multiple imputation).
Handling of withdrawals and lost to follow up	20a	☑	Detailed description of the procedures to be followed when a participant ceases participation in the study prematurely or is lost to follow up
Replacements	20b	☑	Plans and methods of the replacement or substitution of withdrawn participants.
Outcome	21	☑	Definition and description of all primary, secondary and other outcomes.
Data confidentiality statement	22	☑	A detailed description of process to ensure data confidentiality.
Follow up	23	☑	A detailed plan of follow up including schedule and methods (telephonic, house based, hospital based etc.) of follow up.
Plan of study monitoring	24	☐ NA	Description of plan for study monitoring and whether the monitoring will be independent from investigators or sponsors.
Training of surveyors/data collectors	25	☑	Description of how investigators and surveyors will be trained to conduct the research activity.
Quality assurance	26	☑	Plan of quality assurance. back-checking data collection.
**Part D: Ethical consideration**			
Ethical approval	27a	☑	Plan for seeking ethics approval from ethics committees/institutional review boards. If known, give name of ethical committees.
	27b	☐NA	If ethics approval will not be sought, give justification.
Consent and assent	28a	☐ NA	Description of who will obtain informed consent or assent from potential study participants or authorized surrogates, and how (e.g., written informed consent, verbal consent, video/audio recording of consent procedure etc.)
	28b	☑	Give reason if consent or assent not sought.

Section / Item	Item Number		Description
	28c	☐ NA	Give reference to where informed consent forms and applicable translations plan can be found, if not in the protocol.
Risk/harm to participants	29a	☑	A detailed description of potential risks or harms to study participants.
	29b	☑	Plans for collecting, assessing, reporting, and managing any study procedures related adverse events (e.g. adverse events due to blood collection) and other unintended effects of study conduct (e.g. risk to breach confidential and sensitive information of participants)
	29c	☑	Give a statement about whether data will be anonymous, pseudonymized, or can be directly linked to participants.
	29d	☐ NA	Description of any plan for giving Incentives to the participants
Adverse event and serious adverse event reporting	30	☑	Outline how adverse events and serious adverse events information will be collected and reported.
Involvement of patient/participant representatives in protocol development	31	☑	Patient and Public Involvement (PPI) statement including how patients or participants involved in the planning of the study. Give statement, if there is no plan to involve of patient/participants and public in designing or any phase of the study
**Part E. Reporting and dissemination**			
Dissemination/ publication plan	32a	☑	Plans for investigators and sponsor to communicate study results to ethical review boards, participants, key stake holders, the public, and other relevant groups.
	32b	☑	Methods to communicate findings (e.g., via publication (open access or closed access), reporting in results databases, or other data-sharing arrangements), including any publication restrictions.
	32c	☑	Define authorship eligibility guidelines (e.g., ICMJE recommendations)
**Part F: Others**			
Whether Artificial Intelligence (AI) assisted technology was used in writing the protocol	33	☑	Disclose whether authors used artificial intelligence (AI)-assisted technologies in the production of protocol (e.g., chatbots) or there is planning to use artificial intelligence (AI)-assisted technologies in the production of manuscript or study reports.
	34	☐ NA	Give the name of AI tools (such as ChatGPT). Include a statement if authors did or did not review and edited the content created by AI-assisted technologies
References	35	☑	A complete list of references cited in protocol.
Funding	36	☑	Source of any funding for the study and the role of the funders for the study
Open science	37a	☐ NA	**Registration of observational study:** Study identifier and registry name (e.g., open science framework,

Section / Item	Item Number		Description
			ClinicalTrials.gov, ICTRP or any other national or international study registry platform). If not yet registered, name of intended registry.
	37b	☐	**Data sharing:** Plans, if any, for granting public access to the (1) full protocol and amendments, (2) participant-level data set, (3) Statistical analysis plan, (4) statistical codes and other study material (e.g., case report forms, study questionnaires and Informed consent forms). Give reference to where these documents can be found, if not included as annex in the protocol.
